# The Computational Fluid Dynamics (CFD) Analysis of the Pressure Sensor Used in Pulse-Operated Low-Pressure Gas-Phase Solenoid Valve Measurements

**DOI:** 10.3390/s21248287

**Published:** 2021-12-11

**Authors:** Dariusz Szpica, Grzegorz Mieczkowski, Andrzej Borawski, Vitalis Leisis, Saulius Diliunas, Tilmute Pilkaite

**Affiliations:** 1 Faculty of Mechanical Engineering, Bialystok University of Technology, 45C Wiejska Str., PL-15351 Bialystok, Poland; g.mieczkowski@pb.edu.pl (G.M.); a.borawski@pb.edu.pl (A.B.); 2 Faculty of Mechanical Engineering and Design, Kaunas University of Technology, 56 Studentų Str., LT-50240 Kaunas, Lithuania; vitalis.leisis@ktu.lt (V.L.); saulius.diliunas@ktu.lt (S.D.); tilmute.pilkaite@ktu.lt (T.P.)

**Keywords:** mechanical engineering, supply system, computational fluid dynamics (CFD), pressure sensor, solenoid valve, research

## Abstract

This paper presents a flow analysis of the original pressure sensor used to determine times until full opening and closing of the pulse-operated low-pressure gas-phase solenoid valve. The sensor in question, due to the fast variation of the process lasting several milliseconds, has high requirements in terms of response time and ability to identify characteristic parameters. A CFD code has been employed to successfully model the flow behavior of the original pressure sensor used to determine times until full opening and closing of the pulse-operated low-pressure gas-phase solenoid valve at different inlet flow conditions, using the Eulerian multiphase model, established on the Euler–Euler approach, implemented in the commercial CFD package ANSYS Fluent. The results of the modelling were validated against the experimental data and also give more comprehensive information on the flow, such as the plunger displacement waveform. The flow calculations were dynamic in nature; therefore, the experimental plunger displacement waveforms were entered as input in the software for dynamic mash implementation. In identifying the times until full opening and closing, the characteristic points of the pressure waveform on the pressure sensor plate were adopted. CFD flow calculations confirmed the accuracy of identifying the times until full opening and closing by relating them to the results from the plunger displacement sensor. The validation of the results of calculations with the analyzed sensor and the original stand also confirmed the correctness of the use of this type of method for the assessment of gas injector operating times. In the case of time until full opening, the CFD calculations were shown to be consistent with experimental tests, with only a few cases where the relative difference with respect to the displacement sensor reached 3%. The situation was slightly worse in the case of time until full closing, where the results of CFD calculations were in agreement with the displacement sensor, while the experimental test stands had a relative difference of up to 21%. It should be remembered that the sensor evaluates times below 5 × 10^−3^ s, and its construction and response time determine the use depending on the adopted level of accuracy.

## 1. Introduction

Recently, hybrid [1] and electric [2,3] propulsion systems have been increasing in transportation modes. There is advanced work on pneumatic engines and air-assisted combustion engines [4,5,6]. Hybrid and electric drives have many advantages, including higher propulsion flexibility as well as reduced emissions of harmful exhaust components [7]. However, the latter is dependent on how electricity is produced, where emissions are in many cases only relocated. The hydrogen cell is projected to be the most future-proof type of propulsion [8,9]. Work on the use of alternative fuels in classical internal combustion engines also continues unabated. These efforts are aimed at reducing CO_2_ emissions [10] by using fuels with lower carbon content. In addition, modifications are carried out in the area of combustion process organization, where controlled auto-ignition (CAI)/homogeneous charge compression ignition (HCCI) [11,12], high-pressure direct injection (HPDI), or reactivity-controlled compression ignition (RCCI) [13] can be mentioned as main groups. Work is also underway to further develop multi-fuel internal combustion engines [14]. However, one uncertainty is still the emission limitations of the Euro 7 standard [15,16]; according to which, perhaps the internal combustion engine as the sole source of propulsion will not be able to meet in the future.

The most common gaseous fuels used in internal combustion engines are liquefied petroleum gas (LPG) and compressed natural gas (CNG). Alternative fuels are included in the European Union Directive (2014/94/EU) [17]. LPG and CNG have applications in car engines [18,19], work machinery [20,21], and ships [22,23]. In land transport, cars powered by spark-ignition (SI) engines most often use LPG vapor phase power systems [24]. Despite the fact that the development of internal combustion engines and the subsequent emission restrictions have directed LPG supply systems towards liquid-phase injection [18], still a large proportion of engines are powered by vapor phase [25]. Some novelty is the attempts to convert SI engines, which are part of a hybrid power train, to run on vapor-phase LPG [26].

Commercially available LPG vapor-phase systems have a very similar design. Each of them includes an executive element in the form of the low-pressure gas-phase injector. The functioning of the system depends mainly on this element, and a quick assessment of its operational state is a great research challenge.

In experimental studies of the low-pressure gas-phase injectors, a flow meter is mostly used and flow characteristics are determined based on volumetric flow rate [27,28]. There are also attempts to use gasoline injector test benches to determine the flow characteristics of gas injectors [29]. However, using a liquid working medium to test gas injectors is not able to properly reflect the flow characteristics. A rather innovative test method for gas injectors is presented in [29], where the so-called ‘fuel tank refill’ method was used to determine the flow characteristics. The method presented there is an indirect method, where in place of a flow meter, fixed volume tanks are used to act as a flow meter. Based on the pressure variations in the tanks and the simplified flow model (lumped method), the flow parameters are identified numerically. In addition to the flow characteristics, it is possible to evaluate the fuel dosage irregularity of the injection units. A simplified method for evaluating fuel dosage irregularity is presented in [30] using U-tube manometers. Knowledge of the flow characteristics is essential for the proper configuration of the gas supply system and directly affects the external parameters and toxicity of the engine exhaust [31,32,33,34]. It is also very often necessary to determine time until full opening and closing of the gas injectors. Indirect non-invasive methods can be used for this purpose in the simplest variant. The course analyzed are voltage and current in the power line [35,36,37], acceleration sensors the corps of the injector [27,36], flow meters in flow line [27,38], and pressure sensors in the outlet nozzle [39]. The pressure sensors in the outlet nozzle are also used to assess fuel dosage non-repeatability [40]. Apart from that, direct methods are used, which, however, require interference in the injector’s executive system in order to install the displacement sensor measuring needle [36,41] or the needle used in image recording with a high-speed camera [42]. A number of optical sensors, including laser sensors, have also been developed and used in research to enable non-contact measurement of spire elevation [43,44,45]. However, needle/plunger conditions preventing access may be an obstacle here.

Knowledge of time until full opening and closing of the gas-phase injectors is very important for the configuration of the gas supply system, as it influences the gas injection angle and requires appropriate corrections in the control algorithm. Therefore, in the software options for the configuration of the gas supply system there are fields for selecting the injector (type/model) [46]. For a more in-depth analysis of the injection process outside the injector, methods used in liquid fuel injector testing can be used. The most common are image analysis methods with high-speed cameras [47,48,49,50], light fluorescence absorption [51,52], heat flow sensors [53], and optical sensors [54,55]. Measurement techniques using optical laser [52,56,57], phase Doppler particle analyzer (PDPA) system [58], tomographic PIV method [59,60,61] are also used. The possibility of using the methods used in liquid fuel injector testing to test gas injectors is determined by the identification procedures and their applicability in gas flow evaluation. It should also be kept in mind that very often gas injectors are tested using air instead of the target gas for safety reasons. Additionally, values for operating parameters such as volumetric flow rate, time until full opening and closing are provided by manufacturers for air. However, a full functional evaluation can be carried out for the target gas.

The computational fluid dynamics (CFD) is one of the most widely used methods for functional evaluation of pressure, flow, and temperature sensors. The calculations are based on the Reynolds averaged Navier–Stokes (RANS) approach. In addition to the quantitative evaluation to validate the calculations, CFD calculations also allow for a qualitative assessment of the flow. The qualitative assessment, very often difficult or impossible to validate, allows for the establishment of guidelines for the installation or design of sensor connections. In [62] an integrated air pressure and flow sensor is presented. In this case, CFD was used to visualize the flow inside the sensor, with performance prediction in Coventorware based on CFD. In the experimental study, a pressure sensitivity of 1.3 V^−3^·mmHg^−1^ (range (0–50) × 10^3^ Pa) was obtained at flow rates of (0...5) L·min^−1^. The analysis of a capacitive fluid flow sensor is presented in [63]. Using CFD, the input parameters in the form of pressure and velocity were changed to investigate the hydrodynamic parameters. Thus, the geometric parameters of the sensor electrode copula were determined considering the capacitive rest.

Very often there is a need to evaluate the impact of sensor development on their measurement capabilities. The estimation of a pressure response function in a dry seal system and a dynamic pressure measurement for monitoring the seal film pressure is presented in [64]. The response function obtained from the analyses helped to correct for the dynamic pressure effects due to damping. The length of the flexible connection hose was determined to be 115 mm, and the seal spiral grooves were optimized for an inlet angle of 15° and 11°. The calculations are also carried out within separate sections of one sensor. In [65], the effect of separation between two parts of a highly sensitive airflow sensor was analyzed. As a result, the measurement accuracy was improved over conventional measurement systems. CFD calculations show their applicability also with analysis of liquid–liquid flows with high viscosity coefficient. In [66], OpenFOAM and a one-dimensional model were used for CFD, while the RANS model was used for turbulence. Validation of the calculations showed good agreement for pressure drop, volume fractions, and phase distribution. Additionally, the agreement of CFD results with experimental pressure and temperature studies is presented in [67], where the analyses focused on a blunt cone (30 apex-angle and 51 mm base diameter) at wind flow speeds of Mach 6.5 and 8.35 (300 mm hypersonic wind tunnel). In the experimental study using fiber Bragg grating (FBG). The analysis of flow mechanisms related to its aggressiveness was presented in [68]. CFD calculations were validated using piezoelectric polyvinylidene fluoride (PVDF) probes by varying operating frequencies and gaps and shielding. A functional analysis of a sensor built similar to a Prandtl tube is presented in [69]. The analysis of the main sensor operating parameters inferred from the CFD calculations was confirmed in the course of wind tunnel experiments.

Some of the analyses also focus on automotive technology. Trends set by downsizing, downspeeding and the increase in the compression ratio resulted in the evaluation of the influence of selected factors on the combustion process. In [70], using RANS in CFD, the combustion process was analyzed and reflected in the pressure trace recorded by the sensor. The statistical analysis of the indices proposed in the study allowed predicting the possibility of knocking combustion. Moreover, numerical prediction of exhaust emissions has become a certain computational direction in the automotive industry. In [71], a model for predicting nitrogen oxides (NO_x_) emissions in a diesel engine was presented using CFD and a physical model. By validating the calculations with experimental studies, the applicability of the former was demonstrated. It was also found that the proposed predictive model is applicable to 1-D simulations (GT-SUITE, and AMESIM) giving similar results to 3-D CFD. The use of CFD to analyze in automotive lambda sensor pressures allowed the estimation of the sensor protective wall life [72]. Modifications to the probe design reduced the inlet pressure by 1.4%, outlet pressure by 9.8%, exhaust gas velocity by 19.37% and 17.1% respectively, and temperature inside the measurement chamber by 15%. In conclusion, it was found that the proposed modifications will increase its lifetime. Combining CFD (RANS) models with semi-empirical aeroacoustic models is presented in [73], where the performance of automotive axial fans was analyzed to reduce airflow noise. The applicability of the proposed model description was demonstrated for frequencies of (100–2000) Hz as well as (2–10) kHz.

Simulations using CFD are also applied to the analysis of microflow sensor in medicine. The microflow sensor, proposed in [74], was numerically investigated for the effect of its placement in different areas of the human aorta on blood flow characteristics. A 10% increase in blood flow velocity was demonstrated in a cross-section of sensor placement. The presence of the catheter caused a decrease in blood pressure to 768 Pa, and less than 30% of the arterial cross-sectional area experienced an increase in temperature.

The aim of this study was a CFD flow analysis of an original pressure sensor used to determine times until full opening and closing of the low-pressure gas-phase solenoid injector. Literature reports indicate the possibility of using this type of sensor as an indirect method for experimental determination of times until full opening and closing [28,36,39] and dosage non-repeatability [40]. However, there is a lack of flow analysis, where it would be possible to use dedicated software to determine the pressure waveform on the measuring surface of the sensor during dynamic opening and closing of the solenoid valve. Based on the pressure waveform, times until full opening and closing could be determined in a non-invasive way, without interference with the operating system, i.e., the plunger. The validation of the CFD calculation results with the experimental results allowed the evaluation of the measurement capability of the presented sensor and the indication of obtained discrepancies, which should be taken into account when using it.

## 2. Materials and Methods

### 2.1. The Analysed Sensor

**Figure 1:**
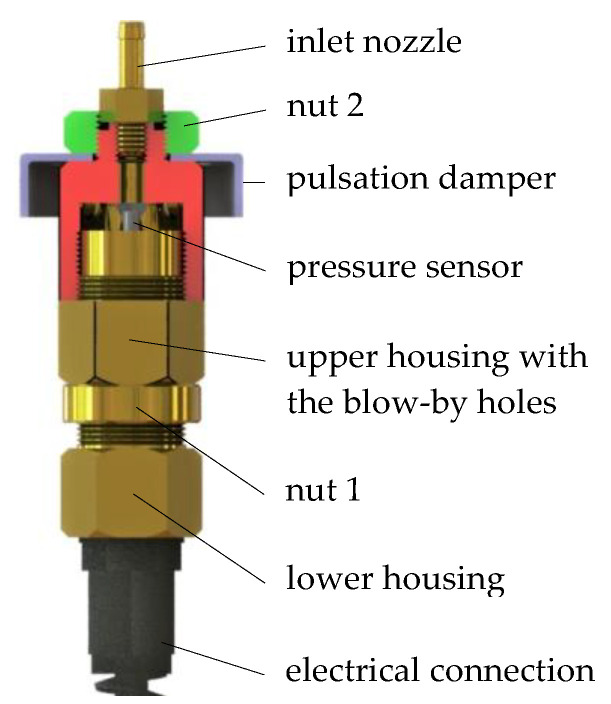
The CAD model of the analyzed sensor.

The object of analysis was the original sensor (Figure 1) used for measuring the pressure at the outlet of a low-pressure gas-phase injector. The main task of this sensor is to measure the rapidly varying pressure at the outlet of a pulsed solenoid valve nozzle. Based on the pressure waveform recorded during the opening and closing of the solenoid valve, the values of functional parameters such as times until full opening and closing can be determined. Apart from times until full opening and closing, the uniqueness of successive opening cycles can also be determined using the area under the pressure curve or the average pressure in a cycle. Measurement capabilities of the presented sensor in experimental conditions have been presented by the author in many publications, including [28,39].

The operation of the sensor is as follows. When the tested low-pressure solenoid valve is opened, air flows through the inlet nozzle into the blow-by hole in the upper housing. In the lower housing a pressure sensor MPXH6400A was mounted. The blow-by holes in upper housing were intended to allow air to flow out of the measuring chamber with the pressure sensor. In the absence of the blow-by holes, the pressure in the chamber would continue to rise, which would prevent the sensor from functioning properly. It was possible to move the pressure sensor using the lower-upper housings threaded connection. Nut 1 was used to lock the threaded connection. Nut 2 was used to attach the pulsation damper. In Figure 1 only part of the pulsation damper is shown due to the large size of the other components. At the bottom of the sensor was the electrical connection for connecting to a power source and sending voltage to the recording device. The power source was a 12 V car battery, which helped to reduce noise during the measurement. An oscilloscope was used to record the courses. The description of the sensor’s measurement capabilities will be presented later in this paper.

The technical data of the MPXH6400A pressure sensor are presented in Table 1.

### 2.2. The Tested Low-Pressure Gas-Phase Solenoid Valve

**Figure 2:**
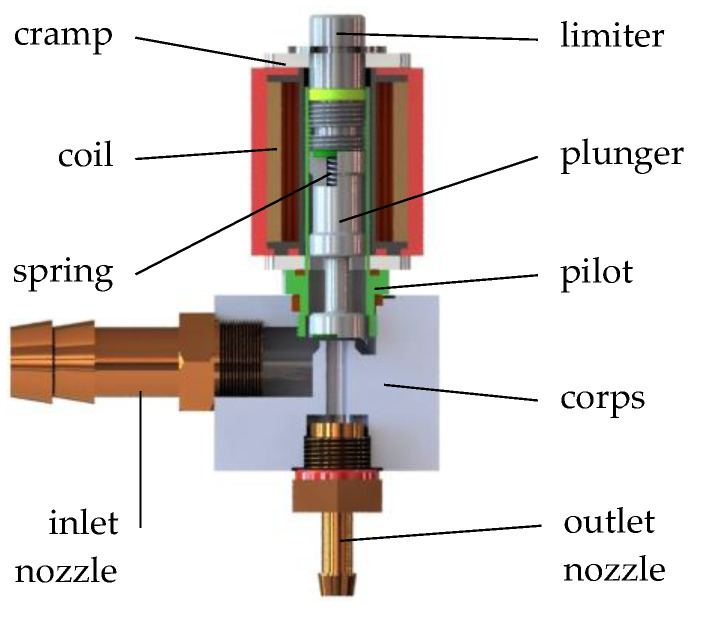
The CAD model of the tested solenoid valve (Valtek Rail Type-30 impulse gas-phase injector).

In order to analyze the performance of the sensor shown in Figure 1, the Valtek Rail Type-30 low-pressure gas-phase injector (Figure 2) was taken as the tested electro valve. This is one of the most popular gas injectors allowing pulse operation. In addition, it allowed easy installation of a displacement sensor to determine the displacement of the piston during the opening and closing phases.

The operation of the solenoid valve is as follows. In the state without electric power, the solenoid valve is closed, the plunger is pressed against the corps by the spring. When an electrical pulse is applied to the coil terminals and the solenoid circuit is closed with the cramp, the plunger overcomes the spring force and the solenoid valve opens. Air begins to flow from the inlet nozzle toward the outlet nozzle. The plunger moves until it contacts the limiter (the limiter sets the maximum stroke). When the electrical impulse at the coil terminals disappears, the electromagnetic field disappears, and the plunger moves toward closure pressed by the spring. The plunger on the corps side and the limiter are equipped with rubber elements to dampen impacts.

The technical data of the tested injector Valtek Rail Type-30 are presented in Table 2.

### 2.3. The Flow Analysis

Ansys Fluent software was used to carry out flow analysis of the gas injector–pressure sensor system under study. This software is widely used in the evaluation of flow processes. In the case presented in this paper, the Reynolds average Navier–Stokes (RANS) approach [77] was used. In this approach, the Reynolds distribution model is substituted into the Navier–Stokes equation resulting in continuity and momentum equations of the form Equations (1) and (2) for incompressible flow:

(1) $$\frac{\partial u_{i}}{\partial x_{i}}=0,$$

(2) $$\rho \left(\frac{\partial u_{i}}{\partial t}+\frac{\partial u_{i}u_{j}}{\partial x_{j}}\right)=-\frac{\partial p}{\partial x_{i}}+\frac{\partial }{\partial x_{i}}\left(\mu \left(\frac{\partial u_{i}}{\partial x_{j}}+\frac{\partial u_{j}}{\partial x_{i}}-\frac{2}{3}\delta_{ij}\frac{\partial u_{l}}{\partial x_{l}}\right)\right)+\frac{\partial }{\partial x_{i}}\left(-\rho \bar{u_{i}‘\;\;u_{j}‘}\right).$$

where $u_{i}$ and $u_{j}$ are the mean velocities; $u_{i}‘$ and $u_{j}‘$ are the fluctuating part of the velocity; p is the dynamic pressure; $\delta_{ij}$ is the Kronecker delta; and ρ is the fluid density.

The last term in brackets in Equation (2) represents the Reynolds stress. As this is unknown, the equations are open. The Boussinesq approximation of Equation (3) is used to solve this problem:

(3) $$-\rho \bar{u_{i}‘\;\;u_{j}‘}=\mu_{t}\left(\frac{\partial u_{i}}{\partial x_{j}}+\frac{\partial u_{j}}{\partial x_{i}}\right)-\frac{2}{3}\left(\rho k+\mu_{t}\frac{\partial u_{k}}{\partial x_{k}}\right)\delta_{ij},$$

where $\mu_{t}$ is the turbulent viscosity; k is the kinetic energy of turbulence.

The turbulent viscosity $\mu_{t}$ can be obtained by solving additional transport equations. The number of additional transport equations depends on the turbulence model chosen. In this study, the *k*-*ω* SST model was decided upon. This model combines the *k*-*ε* and *k*-*ω* turbulence models [78]. The transport equations take the form of Equations (4) and (5):

(4) $$\rho \left(\frac{\partial }{\partial t}k+\frac{\partial }{\partial x_{i}}\left(ku_{i}\right)\right)=\frac{\partial }{\partial x_{j}}\left(\Gamma_{k}\frac{\partial k}{\partial x_{j}}\right)+{\tilde{G}}_{k}-Y_{k}$$

(5) $$\rho \left(\frac{\partial }{\partial t}\omega +\frac{\partial }{\partial x_{i}}\left(\omega u_{i}\right)\right)=\frac{\partial }{\partial x_{j}}\left(\Gamma_{k}\frac{\partial \omega }{\partial x_{j}}\right)+G_{\omega }-Y_{\omega }+D_{\omega }.$$

where ${\tilde{G}}_{k}$ and $G_{\omega }$ represent the production terms of k and ω; $Y_{k}$ and $Y_{\omega }$ represent the dissipation terms of k and ω; $\Gamma_{k}$ and $\Gamma_{\omega }$ represents the effective diffusivity of k, and *ω*; *D_ω_* represents the cross-diffusion term.

Analyses conducted by many researchers as well as commercial software developers to assess the applicability of the *k*-*ε* and *k*-*ω* turbulence models to describe flows have led to some general conclusions. The *k*-*ε* model correctly represents the free flow and the boundary layer. It is also characterized by low sensitivity to feed conditions with respect to the quantities describing turbulence. The *k*-*ω* model, on the other hand, is considered to be the one that more closely represents turbulent flow in the near-wall layer. Additionally, it is characterized by high sensitivity with respect to the quantities describing turbulence in free flow. Therefore, in the analyzed case of flow tests of an injector and a pressure sensor, where we have a large range of flow cross-section and length values, it was considered appropriate to combine both turbulence models.

**Figure 3:**
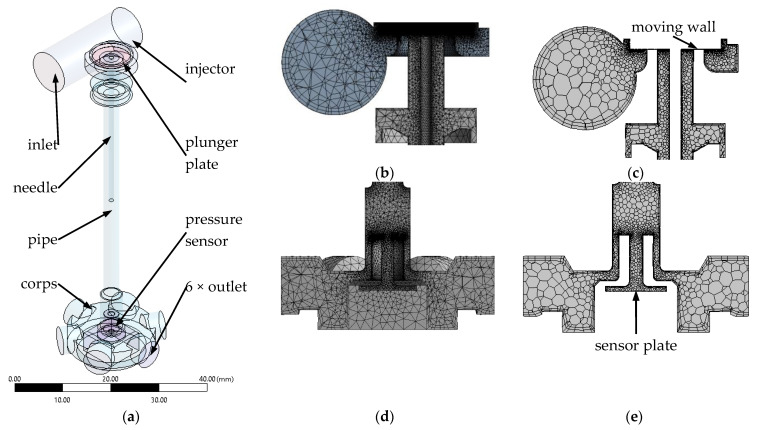
Steps in model conversion: (**a**)—solid model of the fluid; (**b**,**d**)—tetrahedral grid in the upper and lower parts of the study area; (**c**,**e**)—polyhedral grid in the upper and lower parts of the study area.

For the calculations, a solid model of the fluid was created (Figure 3a) including a division into sections where initial and boundary conditions could be determined. The division into sections also made it possible to vary the type and density of the mesh. Of course, the compatibility of the grid geometry at the junctions of the sections was kept in mind. The fluid geometry was created using Ansys Geometry software. It was simplified, omitting all the outlines of the threaded connections that remained in the fluid area. In addition, the area around the injector plunger was limited to only the lower part, which had the greatest influence on the flow character. In order to measure selected hard-to-reach dimensions inside the injector, it was necessary to cut out the body walls, which was possible as several bodies were available. The pressure sensor itself, due to its design, only required the disassembly of the threaded connections to create the fluid geometry. The measurements required to create the fluid geometry used a tool with an accuracy of 0.05 × 10^−3^ m. In calculations it was necessary to leave the firing pin, which was used in experimental tests, in order to best represent the real working conditions of the injector. Of course, in the experimental tests, the analyzed sensor will work with a gas injector without the firing pin, where, of course, time until full opening and closing will be different due to a smaller mass in progressive motion. In order to maintain grid continuity, the injector plunger was offset from the seat by 0.05 × 10^−3^ m, which may cause a small leak, but due to the fast-variable process will not affect the calculation result.

In the initial phase of dividing the area into elements, a tetrahedral mesh was used (Figure 3b,d). In the different parts of the study area the maximum element size was taken as: moving wall (plunger) and side wall of the plunger 0.025 × 10^−3^ m; area under the plunger 0.05 × 10^−3^ m; and sensor plate 0.2 × 10^−3^ m. Two inflation layers and no slip were used on all walls. The global growth rate was set to 1.2. Other mesh parameters were selected systemically by Ansys software. Further using Fluent solving, the tetrahedral mesh was converted to a polyhedral mesh using system options including cell optimization (Figure 3c,e). This was performed in order to reduce the computation time, which as reported in the literature [79,80,81,82], does not significantly affect the result.

A standard SIMPLE scheme with control values of 1 × 10^−4^ residuals was used to solve the flow problem in Ansys Fluent. The discretization of the continuity and momentum equations was carried out using a pressure-based solver. At steady state, the equations can be written in integral form (Equations (6) and (7)):

(6) $$\rho \oint \vec{v}\;\;d\vec{A}=0.$$

(7) $$\rho \oint \vec{v}\vec{v}\;\;d\vec{A}=-\oint pI\;\;d\vec{A}+\oint \bar{\tau }\;\;d\vec{A}+\int_{V}\vec{F}\;\;dV.$$

where I is the identity matrix; $\bar{\tau }$ is the stress tensor; and $\vec{F}$ is the force vector.

Equation (7) is integrated by the control volume resulting in the discrete equation (Equation (8)):

(8) $$\sum_{f}^{N_{\mathrm{faces}}}J_{f}A_{f}=0.$$

where *J_f_* is the mass flux through face f, $\rho v_{n}$.

In a further step, it was necessary to relate the velocity values $v_{n}$ to the surface. The averaging of velocity values over the face is realized by momentum-weighted averaging using the weighting factor $a_{p}$. We then write the frontal flux $J_{f}$ in the form of Equation (9):

(9) $$J_{f}=\rho \frac{a_{p,c_{0}}v_{n,c_{0}}+a_{p,c1}v_{n,c_{1}}}{a_{p,c_{0}}+a_{p,c_{1}}}+d_{f}\left(\left(p_{c_{0}}+{\left(\nabla p\right)}_{c_{0}}{\vec{r}}_{0}\right)-\left(p_{c_{1}}+{\left(\nabla p\right)}_{c_{1}}{\vec{r}}_{1}\right)\right)={\hat{J}}_{f}+d_{f}\left(p_{c_{0}}-p_{c_{1}}\right).$$

where $p_{c_{0}}$ and $p_{c1}$ are the pressure within the two cells on either side of the face; $v_{n,c_{0}}$ and $v_{n,c_{1}}$ are the velocity within the two cells on either side of the face; ${\hat{J}}_{f}$ contains the influence of velocities in these cells; *d_f_* is a function; ${\bar{a}}_{p}$ the average of the momentum equation; and $a_{p}$ coefficients for the cells on either side of face f.

In the area of the moving wall, the parameters smoothing, layering, and remeshing were applied according to the requirements of the software used. Due to the length of the descriptions, they are not presented in the paper. The injectors plunge displacement required for flow studies was obtained from the experimental stand presented later in this paper.

Air was used as the working medium. In the experimental tests of the low-pressure gas-phase injector, it is used for safety reasons. The use of air was considered appropriate in the context of further reference to the experimental results of other gas injectors. Each time, the supply conditions, the inlet–outlet pressure differential, and the corresponding plunger displacement waveform were changed in the calculations.

### 2.4. The Experimental Test Stand

**Figure 4:**
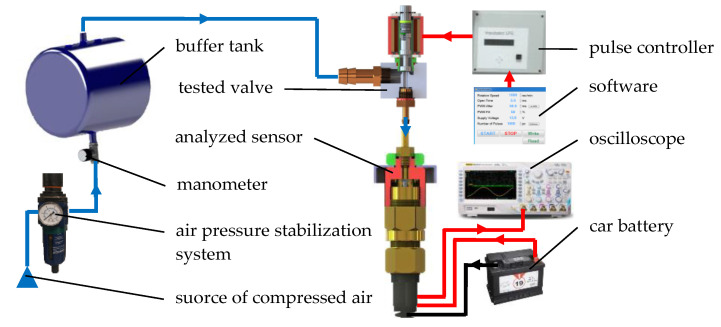
The scheme of the flow test stand.

Validation of the numerical calculations was performed using a gas injector flow test stand. The tests were carried out using air as the working medium similarly to the calculations. The operation of the stand is as follows. Compressed air from the source of compressed air flowed through the air pressure stabilization system and further to the buffer tank Figure 4. The purpose of the buffer tank was to reduce pressure pulsations in the supply system during cyclic operation of the solenoid valve. From the buffer tank, air flowed to the inlet nozzle tested valve (Valtek Rail Type-30), and each time the valve was open, to the analyzed sensor. The cyclic operation of the solenoid valve was controlled by STAG AC LLC based pulse induction system controller with dedicated software. The analyzed sensor was powered with a car battery, while the measurement signal was supplied with a RIGOL MSO4014 oscilloscope. The pressure was recorded with a step of 0.01 × 10^−3^ s.

In order to determine the course of the injector plunger stroke, the stand shown in Figure 4 was supplemented with additional equipment. The needle CL80 ZEPWM displacement sensor (response time: <0.1 × 10^−3^ s, range: ±1 × 10^−3^ m, accuracy: 1%) was mounted on the plunger. The mass of the mounted needle was 0.64 × 10^−3^ kg, which increased the mass of the plunger by 11.26% (ABT-100 KERN scales). The static characteristics of the sensor was determined for which the coefficient of determination on the level of *R*^2^ = 99.97% was obtained. The RIGOL RP1500A (bandwidth ~150 × 10^6^ Hz; damping factor—10:1) was used for the electric voltage measurement, while the HAMEG HZ050 (response time: <0.1 × 10^−6^ s, range: ±30 A, accuracy: 1%) was used for the electric current measurement. Additionally, a KELAG KAS903-02A acceleration sensor (response time: < 1 × 10^−3^ s; repeatability <4 × 10^−3^ g, range: ±12 g, accuracy: 0.005 g) was mounted to the solenoid valve body. Acceleration sensor was to compare the functional parameters (time until full opening and closing) obtained with the displacement sensor. The signals were recorded with a RIGOL MSO4014 oscilloscope (bandwidth—100 × 10^6^ Hz; real-time sample rate—up to 4 × 10^9^ Sa·s^−1^). The registration step was assumed to be 0.01 × 10^−3^ s.

**Figure 5:**
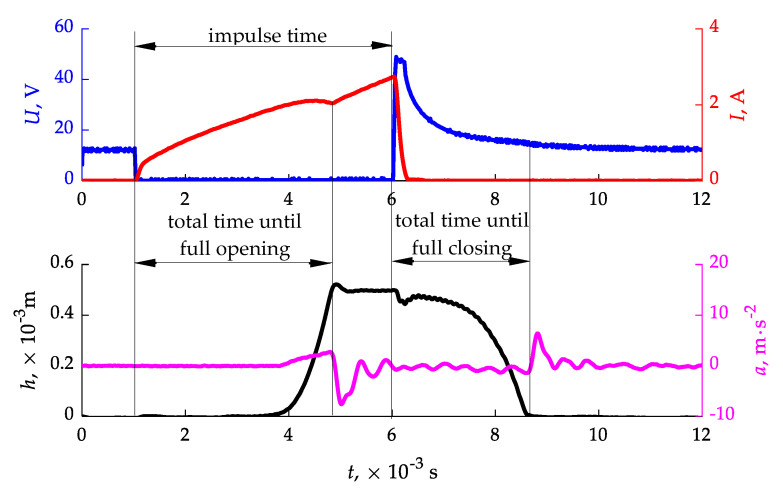
The measurements results: *U*—electric voltage (blue), *I*—electric current (red), *h*—plunger displacement (black), *a*—acceleration of the electro valve corps (magenta).

In the initial stage, the effect of the mass of the firing pin mounted to the plunger on time of opening and closing the injector was evaluated. The tests were carried out at atmospheric pressure *p_a_* = 1 × 10^5^ Pa, and ambient temperature *T_a_* = (20 + 273.15) K. The supply pressure was set at *p_in_* = 1 × 10^5^ Pa, while the injection time (pulse time) at 5 × 10^−3^ s. Figure 5 shows the electric voltage and current, plunger displacement and acceleration of the electro valve corps. Time values read from Figure 5 were: total time until full opening 3.93 × 10^−3^ s and total time until full closing 2.67 × 10^−3^ s, respectively. This increased time until full opening by about 15%, and time until full closing by about 21%, relative to the manufacturer’s declaration (Table 2).

## 3. Results and Discussion

### 3.1. Plunger Lift

**Figure 6:**
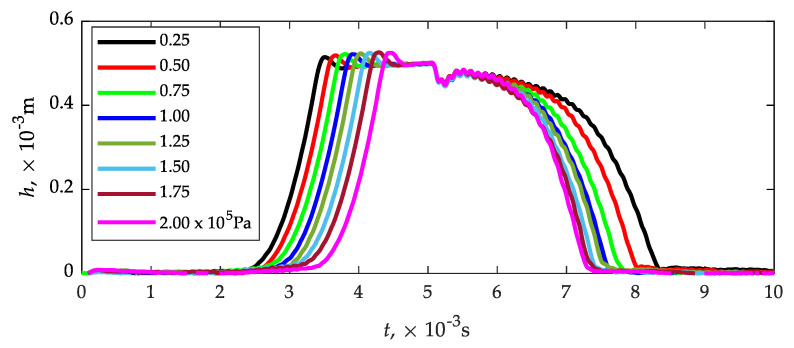
Plunger displacement curves depending on pressure supply (0.25…2.00) × 10^5^ Pa.

Before proceeding to the numerical analysis, it was necessary to determine the plunger lift as a function of time at a given pulse length controlling the opening of the solenoid valve. For this purpose, the flow stand presented in Figure 4 was used. The plunger lift courses were determined at various supply pressures (0.25...2.00) × 10^5^ Pa, step 0.25 × 10^5^ Pa (Figure 6). Time characteristics of the plunger displacement were necessary to initiate the CFD.

In order to reduce the CFD calculation time, the number of plunger displacement measurement points was limited to a time step of 0.1 × 10^−3^ s. Interpolation to the required time step was performed using the cubic spline method. The plunger waveform was loaded into the Ansys Fluent workspace taking into account the other required simulation conditions.

### 3.2. CFD Analysis

In the initial phase of the CFD calculations, the influence of the mesh density on the pressure waveforms obtained at the pressure sensor measuring surface was checked (Figure 3e). For this purpose, four tests were carried out with the input grid parameters given in Table 3. The grid was initially constructed as a tetrahedral mesh, and in the first variant (*mesh 1*), the values proposed by the system were used. In further steps, the mesh was thickened (*mesh 2...4*). The input–output pressure difference was defined as a relative value of 1 × 10^5^ Pa (output—ambient conditions). The calculation time step was defined as 1 × 10^−4^ s. The remaining calculation parameters were adopted in accordance with Section 2.3 of the study. In a further step, in order to reduce the calculation time, the tetrahedral mesh was converted into a polyhedral mesh using Fluent solving with the use of system options, including cell optimization.

**Figure 7:**
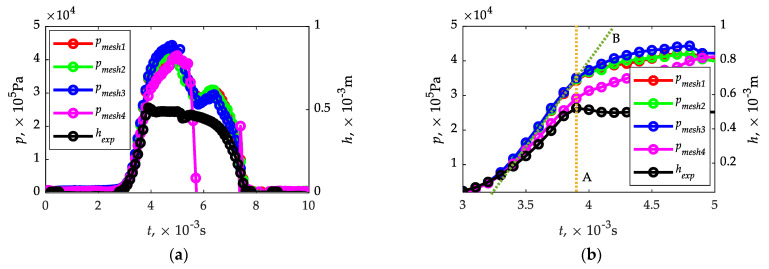
Identification of injector times until full opening and closing from pressure waveforms on the sensor plate: (**a**)—whole waveforms; (**b**)—selected range of waveforms.

In all analyzed cases of mesh density (Table 3), the average quality parameters (skewness and orthogonal quality) should be considered correct [83]. For *mesh 3* and *mesh 4*, the number of elements increased significantly which affected the calculation time. Based on the analysis of the influence of mesh density on the calculation results, it was concluded that the general mesh parameters proposed systemically (*mesh 1* in Table 3), taking into account the local densities described in Section 2.3, are sufficient to identify time until full opening and closing of the gas injector (Figure 7). In the case of *mesh 4* the calculations did not give a correct solution in the range (5.5…7.5) × 10^−3^ s, and the pressure waveform itself differed from the others. The task of the analyzed sensor is to identify times until full opening and closing of the gas injector. The opening pulse is in the range of (0...5) × 10^−3^ s, to which the plunger responds with opening h according to (Figure 7a). While time until full closing at all tested mesh densities (Table 3) is easy to identify (pressure at the sensor drops to 0 Pa), and is 2.8 × 10^−3^ s, the identification of time until full opening becomes a problem. In this case, it is proposed to evaluate time until full opening on the basis of the “inflection” point of the pressure course (Figure 7b). A sharp increase in pressure on the sensor plate corresponds to time until full opening of the injector. On the other hand, a decrease in the incremental gradient signals full opening. All tested mesh (*mesh 1…4*) density variants gave the possibility of identifying times until full opening; therefore, *mesh 1* was selected for further analyses, as in this case the calculations were the least time-consuming. Note that the identification of injector times until full opening and closing is derived from time step (1 × 10^−4^ s) and can be reported with this accuracy.

**Figure 8:**
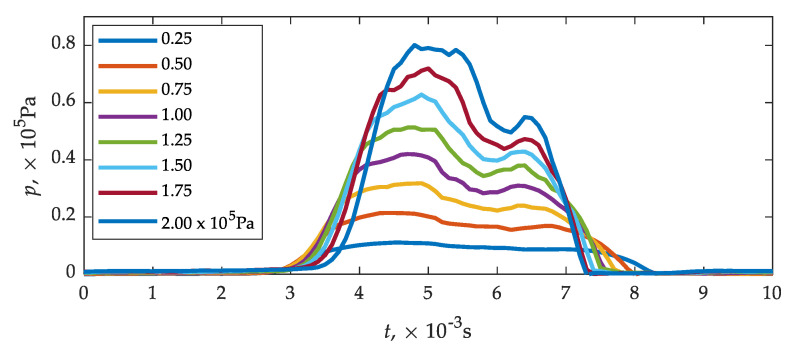
Pressure waveforms on the sensor plate of the analyzed sensor.

The value of the inlet–outlet pressure equation in the range (0.25...2.00) × 10^5^ Pa was assumed as a variable in the calculation. The pressure differential was adjusted by leaving ambient conditions at the outlet. This was dictated by the fact that a pressure sensor was used to evaluate the functional parameters of the injector and its sensitivity to this variable is important. In tests of the low-pressure gas-phase injectors, the pressure difference is usually 1 × 10^5^ Pa, but it was considered important to know a specific range around this value. The obtained waveforms/values may be applicable in modelling transient processes of the engine power system operation, where the pressure difference may be different than 1 × 10^5^ Pa. For the calculations to be correct, it was necessary to determine the experimental plunger lift curves at different inlet–outlet pressures, since they affect the injector times until full opening and closing, as described in [84]. By taking the plunger lift curves (Figure 6) and varying the pressure in the range (0.25...2.00) × 10^5^ Pa, the sensor plate pressure variation curves (Figure 8) were obtained.

The effect of pressure on times until full opening and closing can be seen (Figure 8), for which the plunger lift curve is directly responsible. An increase in pressure causes an increase in time until full opening, which is due to the need to overcome more pressure on the plunger. The pressure has the opposite effect on time until full closing, time decreases.

**Figure 9:**
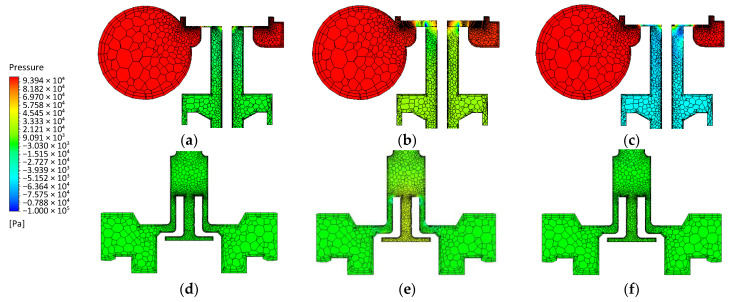
Pressure distributions around the plunger and pressure sensor: (**a**,**d**)—*t* = 2 × 10^−3^ s; (**b**,**e**)—*t* = 4 × 10^−3^ s; (**c**,**f**)—*t* = 7.5 × 10^−3^ s.

Figure 9 shows the pressure distributions of selected zones of the tested system, near the plunger and the pressure sensor (pressure difference 1 × 10^5^ Pa). In the initial phase (*t* = 2 × 10^−3^ s), the pressure difference caused by the initial simulation conditions is visible, while the maximum pressure occurs in the injector area. The need to maintain the continuity of the grid resulting in a gap between the plunger plate and the seat of 0.05 × 10^−3^ m does not substantially affect the pressure distribution in the zone behind the plunger plate (Figure 9a,d). At the maximum opening of the injector (*t* = 5 × 10^−3^ s), the flow of air from the injector to the duct and further to the pressure sensor causes an increase in pressure at the sensor plate (Figure 9b,e). When the injector is closed (*t* = 7.5 × 10^−3^ s), there is negative pressure in the area under the plunger plate, but this is not reflected in the sensor plate (Figure 9c,f).

### 3.3. Validation of the CFD Calculation Results

**Figure 10:**
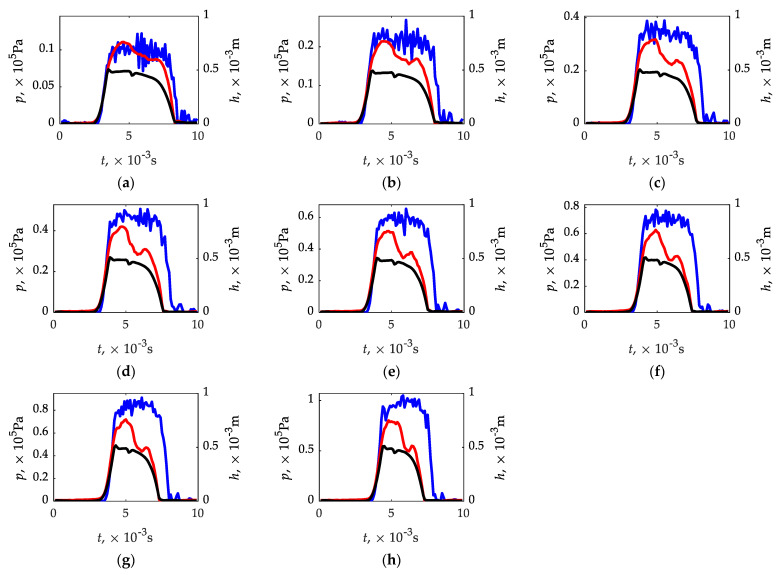
Validation of the CFD calculation results. Legend: (**a**)—0.25 × 10^5^ Pa; (**b**)—0.25 × 10^5^ Pa; (**c**)—0.25 × 10^5^ Pa; (**d**)—0.25 × 10^5^ Pa; (**e**)—0.25 × 10^5^ Pa; (**f**)—0.25 × 10^5^ Pa; (**g**)—0.25 × 10^5^ Pa; (**h**)—0.25 × 10^5^ Pa; —displacement sensor (black); —pressure from CFD calculation (red); —experimentally determined pressure (blue).

In the final stage of the analysis, the results of the CFD calculations were validated. For this purpose, the analyzed sensor was mounted on the test stand presented in Section 2.4. By varying the inlet pressure in the range of (0.25...2.00) × 10^5^ Pa at a fixed outlet pressure (ambient conditions), experimental tests were carried out, which were compared with the waveforms calculated using CFD (Figure 10). The identification of times until full opening and closing was referred to the plunger lift determined in the earlier study. The increasing difference in the maximum values of pressure obtained from the CFD calculations and those obtained from the experimental tests of the analyzed sensor may result from the simplification that was made by creating a solid model of the fluid. It was assumed in the calculations that the output pressure would be the averaged pressure with the sensor plate at the rigid wall (Figure 3). In the measurement system of the actual sensor, there is a diaphragm that acts on the piezoelectric sensor. Due to the deformation of the diaphragm, it acts with a certain delay when the pressure rises, as can be seen in the experimental runs (Figure 10). The higher the pressure, which flows from the injector to the sensor, the faster the diaphragm deformation and, as a result, the impact, which can be seen in the form of a peak. On the other hand, when the pressure drops, in addition to the deformation of the diaphragm, it is necessary to remove air from the measuring chamber, which can be the reason for the delay in operation when evaluating the time until full closing. In the comparative analysis of CFD calculations and experimental tests, it should also be borne in mind that the tests are of a fast-variable character, and the response time declared by the sensor manufacturer at the maximum level of 1 × 10^−3^ s may appear both at the increase and decrease in pressure. Apart from looking for the reasons of discrepancies between the results of CDF calculations and experimental investigations on the side of sensor operation and replacement of the diaphragm with a rigid reference wall, one should also take into account the imperfection of the mathematical model as well as simplifications in the solid model of the fluid. The k-ω SST model used in this study, which combines features of the *k*-*ε* and *k*-*ω* models, may not sufficiently represent the nature of the flow. The flow in the area of gas injector—pressure sensor is characterized by high complexity due to differentiation of flow cross-section and fast variability of the process. The dynamic rebuilding of the mesh created when the injector is opened may also be here for a reason. However, the most likely cause of the differences is the susceptible diaphragm in the sensor measurement system, which essentially delays the identification of the closing process due to the need to remove air.

It can be seen (Figure 10) that there is agreement in times until full opening and closing comparing CFD calculations and plunder displacement. As for times until full opening in relation CFD calculations—experimental tests of the sensor, they agree for (0.75; 1.00; 1.25, and 1.75) × 10^5^ Pa, in other cases (0.25; 0.50; 1.50, and 2.00) × 10^5^ Pa the differences do not exceed 3% (Table 4 and Table 5). On the other hand, the times until full closing determined from CFD calculations and the displacement sensor are in agreement (Table 4 and Table 5). The situation is slightly worse in the relation between CFD calculations and experimental tests of the analyzed sensor. In this case, CFD calculations are characterized by significant discrepancies. Only for the differential pressure of 0.25 × 10^5^ Pa the relative error is below 10%, for the rest it reaches 21% (Table 5).

**Figure 11:**
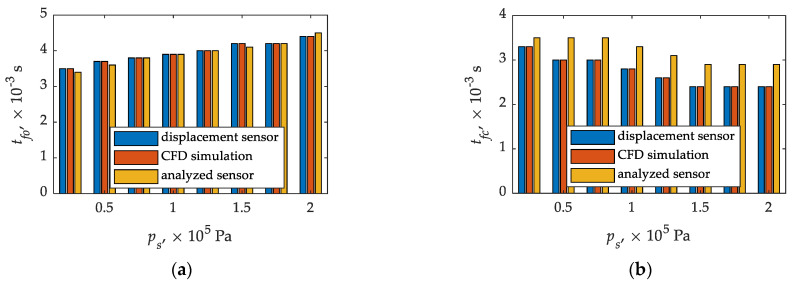
Influence of inlet–outlet pressure difference on injector times until full opening and closing. Legend: (**a**)–until full opening; (**b**)–until full closing.

On the basis of the obtained results of CFD calculations and experimental tests, the influence of the inlet–outlet pressure difference on the times until full opening and closing of the gas injector Figure 11 was confirmed, which was signaled for a different injector design solution in [84]. Apart from an inlet–outlet pressure difference of 0.25 × 10^5^ Pa, the times until full closing determined experimentally differ significantly from those obtained from CFD calculations (Figure 11).

**Figure 12:**
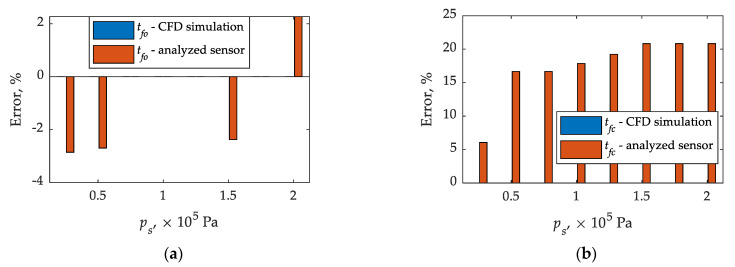
Relative differences in times until full opening and closing obtained from CFD calculations and experimentally. Legend: (**a**)–until full opening; (**b**)–until full closing.

The relative differences in the identified times until full opening and closing of the gas injector, presented in Figure 12, can be attributed to simplifications in the creation of the solid model of the fluid, indicated earlier in the paper, which do not fully reflect the measurement capabilities of the actual sensor.

## 4. Conclusions

The aim of the study was a CFD flow analysis of an original pressure sensor used to determine times until full opening and closing of the low-pressure gaseous phase solenoid valve. Despite the ever-increasing share of direct injection engines, alternative power systems using indirect LPG injection are among the most popular in transport. The evaluation of a fast-changing process such as gas injection can be performed by many methods. In the study, an original pressure sensor was proposed, with the use of which the evaluation times until full opening and closing is possible. The evaluation of these times uses the pressure waveforms and their interpretation. In the initial stage, a simplified solid model of the fluid was created and then a finite element mesh was applied. Using the dynamic mesh generation capabilities of Ansys Fluent software, calculations were carried out to determine the pressure waveform on the sensor plate during the opening and closing of the gas injector. The plunger displacement waveforms required for the calculations were determined experimentally. In the final stage, the CFD results were validated using the real object and the original test bench.

On the basis of the conducted research, it has been stated:

- The proposed simplified solid fluid model allowed to reflect the flow capabilities of the investigated gas injector—pressure sensor system.
- The analyzed mesh densities in a global view together with local user definitions showed differences in the runs. In all mesh cases, the mean quality parameters, skewness (0.22), and orthogonal quality (0.86) were of good quality. Each of the presented mesh densities gave the possibility to identify the times until full opening and closing of the injector, so it was decided to use the system-proposed mesh with local user definitions. Due to time-consuming calculations, the final models used a polyhedral mesh built systemically with cell optimization.
- Experimental studies using a plunger displacement sensor showed a 15% increase in time until full opening and 21% increase in time until full closing. This was due to the use of a needle in the injector operating system. The results were necessary to initiate a dynamic mesh in Ansys Fluent. The needle was left in CFD calculations and validation on the experimental bench.
- The indirect method of evaluating times until full opening based on the pressure waveform on the pressure sensor plate had a positive effect. The drop in the pressure rise gradient coincided with the maximum lift determined using the plunger displacement sensor.
- The indirect method of evaluating times until full injector closing, when pressure at the pressure sensor plate drops to 0 Pa, is also able to represent the end of the injector closing process.
- The times until full opening of the injector determined using plunger displacement sensor, CFD simulation, and pressure plate sensor give similar results. No differences were found at inlet–outlet pressure differences (0.75; 1.00; 1.25, and 1.75) × 10^5^ Pa by relating the pressure waveforms to the displacement sensor. In the remaining cases (0.25, 0.50, 1.50, and 2.00) × 10^5^ Pa, the differences did not exceed 3%;
- In the case of times until full closing of the injector in the relation CFD calculations—experimental studies, only for the pressure difference of 0.25 × 10^5^ Pa the relative error is below 10%, in the remaining cases it reached 21%. The simplification of the solid model of the fluid on the one hand, and the reaction time of the real object on the other, may be responsible for such a significant difference;

The performed tests confirmed the functional correctness of the analyzed pressure sensor. Possible reasons for differences in the determined times of until full opening and closing of the injector were explained. At a later stage, we plan to make an original connection, which will bring the pressure sensor as close as possible to the injector valve, which should positively influence the evaluation of the times in question.

## Figures and Tables

**Table 1:** The technical data of the MPXH6400A sensor [75].

Parameter	Value
Nozzle size	4 × 10^−3^ m
Pressure range	(20...400) × 10^3^ Pa
Max. pressure	1600 × 10^3^ Pa
Storage temperature	(−40…+125) + 273.15 K
Supply voltage	(4.64…5.36) V
Supply current	3 × 10^−3^ A
Accuracy (0…85 °C)	±1.5%
Sensitivity	12.1 (10^−3^ V / 10^3^ Pa)
Response time	<1.0 × 10^−3^ s
Warm-up time	20 × 10^−3^ s
Offset stability	±0.25% V

**Table 2:** The technical data of the Valtek Rail Type-30 injector [76].

Parameter	Value
Nozzle size	4 × 10^−3^ m
Piston stroke	0.4 × 10^−3^ m
Coil resistance	3 Ω
Time until full opening	3.4 × 10^−3^ s
Time until full closing	2.2 × 10^−3^ s
Max. working pressure	4.5 × 10^3^ Pa
Operating temperature	(−20…+120) + 273.15 K

**Table 3:** The grid parameters adopted in the initial part of the calculation.

Variant	Min Size, mm	Max Face Size, mm	Max Face Size, mm	Curvature Normal Angle, deg	Growth Rate	Elements	Skewness	Orthogonal Quality
mesh 1 *	0.01	1.22	2.45	18	1.2	2,353,288	0.2264	0.8675
*mesh 2*	0.01	1.00	2.00	18	1.2	2,357,922	0.2265	0.8647
*mesh 3*	0.01	0.25	0.50	18	1.2	3,282,392	0.2249	0.8679
*mesh 4*	0.01	0.10	0.20	18	1.2	13,064,272	0.2151	0.8708

* System proposal.

**Table 4:** Times until full opening and closing of the gas injector identified using different methods.

**Method**	**Time,** **× 10^−3^ s**	***p_s_* × 10^5^ Pa**							
		0.25	0.50	0.75	1.00	1.25	1.50	1.75	2.00
displacement sensor	*t_tfo_*	3.50	3.70	3.80	3.90	4.00	4.20	4.20	4.40
	*t_tfc_*	3.30	3.00	3.00	2.80	2.60	2.40	2.40	2.40
CFD simulation	*t_tfo_*	3.50	3.70	3.80	3.90	4.00	4.20	4.20	4.40
	*t_tfc_*	3.30	3.00	3.00	2.80	2.60	2.40	2.40	2.40
experimentally	*t_tfo_*	3.40	3.60	3.80	3.90	4.00	4.10	4.20	4.50
	*t_tfc_*	3.50	3.50	3.50	3.30	3.10	2.90	2.90	2.90

**Table 5:** Relative difference in the injector times until full opening and closing determined with respect to the displacement sensor.

**Method**	**Relative** **Difference, %**	***p_s_* × 10^5^ Pa**							
		0.25	0.50	0.75	1.00	1.25	1.50	1.75	2.00
CFD simulation	Δ*t_tfo_*	0.00	0.00	0.00	0.00	0.00	0.00	0.00	0.00
	Δ*t_tfc_*	0.00	0.00	0.00	0.00	0.00	0.00	0.00	0.00
experimentally	Δ*t_tfo_*	−2.86	−2.70	0.00	0.00	0.00	−2.38	0.00	2.27
	Δ*t_tfc_*	6.06	16.67	16.67	17.86	19.23	20.83	20.83	20.83

## Data Availability

Data sharing not applicable.
